# Synthesis and Bioinformatic Characterization of New Schiff Bases with Possible Applicability in Brain Disorders

**DOI:** 10.3390/molecules26144160

**Published:** 2021-07-08

**Authors:** Speranta Avram, Ana Maria Udrea, Diana Camelia Nuta, Carmen Limban, Adrian Cosmin Balea, Miron Teodor Caproiu, Florea Dumitrascu, Cătălin Buiu, Alexandra Teodora Bordei

**Affiliations:** 1 Department of Anatomy, Animal Physiology, and Biophysics, Faculty of Biology, University of Bucharest, 36-46 M. Kogălniceanu Boulevard, 050107 Bucharest, Romania; speranta.avram@gmail.com; 2 National Institute of Laser, Plasma and Radiation Physics, 409 Atomistilor Str., 077125 Magurele, Romania; ana.udrea@inflpr.ro; 3 Department of Pharmaceutical Chemistry, Faculty of Pharmacy, “Carol Davila” University of Medicine and Pharmacy, 6 Traian Vuia Street, 020956 Bucharest, Romania; diana.nuta@umfcd.ro (D.C.N.); carmen.limban@umfcd.ro (C.L.); adrian-cosmin.balea@rez.umfcd.ro (A.C.B.); alexandra.telehoiu@drd.umfcd.ro (A.T.B.); 4 The Organic Chemistry Center of Romanian Academy “C. D. Neniţescu”, Splaiul Independenţei 202B, 060023 Bucharest, Romania; dorucaproiu@gmail.com (M.T.C.); fdumitra@yahoo.com (F.D.); 5 Department of Automatic Control and Systems Engineering, Politehnica University of Bucharest, Spl. Independenţei 313, 060042 Bucharest, Romania

**Keywords:** Schiff bases, carbazol derivatives, microwave synthesis, bioinformatics, neuropsychiatric disorders

## Abstract

(1) Background: The research aims to find new treatments for neurodegenerative diseases, in particular, Alzheimer’s disease. (2) Methods: This article presents a bioinformatics and pathology study of new Schiff bases, (*EZ*)-N′-benzylidene-(2*RS*)-2-(6-chloro-9*H*-carbazol-2-yl)propanehydrazide derivatives, and aims to evaluate the drug-like, pharmacokinetic, pharmacodynamic and pharmacogenomic properties, as well as to predict the binding to therapeutic targets by applying bioinformatics, cheminformatics and computational pharmacological methods. (3) Results: We obtained these Schiff bases by condensing (2*RS*)-2-(6-chloro-9*H*-carbazol-2-yl)propanehydrazide with aromatic aldehydes, using the advantages of microwave irradiation. The newly synthesized compounds were characterized spectrally, using FT-IR and NMR spectroscopy, which confirmed their structure. Using bioinformatics tools, we noticed that all new compounds are drug-likeness features and may be proposed as potentially neuropsychiatric drugs (4) Conclusions: Using bioinformatics tools, we determined that the new compound **1e** had a high potential to be used as a good candidate in neurodegenerative disorders treatment.

## 1. Introduction

Neurodegenerative disorders cause progressive degeneration of the structure and function of the central nervous system or peripheral nervous system, and with the population ageing, they have become a topic of interest to the medical world. Common neurodegenerative diseases include Alzheimer’s disease and Parkinson’s disease.

Alzheimer’s disease is characterized by the irreversible destruction of neurons, which leads to the gradual loss of memory and cognitive abilities, which is associated with other symptoms and neuropsychic signs such as depression, apathy, anxiety, agitation and hallucinations. It affects about 2% of the population of industrialized countries. More than 10% of these people are over 65 years old, and 50% are over 85. The number of people who have Alzheimer’s doubles every 20 years, with the total number of patients likely to exceed 80 million by 2040 [1]. The disease is associated with numerous pathophysiological factors, such as extracellular β-amyloid deposits leading to cerebral amyloid angiopathy and inflammation, aggregation of tau proteins in the form of intraneuronal neurofibrillary formation, oxidative stress and low acetylcholine levels. Other risk factors are ApoE glycoprotein, hypercholesterolemia, various environmental agents such as aluminium, mercury and iron ions, pesticides, dietary factors or brain damage of multiple causes [2]. Alzheimer’s disease is of great interest in terms of diagnosis, mechanism of occurrence, and especially treatment, and is a medical crisis that involves many human and financial resources. Currently, drugs aimed at symptomatic therapy are used, and they increase central cholinergic function or decrease glutamatergic excitotoxicity. These correct behavioural manifestations, but recent studies are focused on solutions to reduce the production of pathogenic factors in the brain and to discover drugs to act in the developing stages of the disease, towards the discovery of specific biomarkers and implicitly of targeted therapies to stop the evolution of this form of dementia.

To stop the progression of the disease, treatments should interfere with the links responsible for the appearance of signs and symptoms. Studies have shown that there is a relationship between β-amyloid plaques and low brain glucose use in Alzheimer’s patients. In patients with moderate behavioural manifestations or familial Alzheimer’s risk, carbohydrate hypometabolism occurs before neurodegeneration and neuronal atrophy. [3]. For anti-amyloid treatment, passive immunotherapy with monoclonal antibodies has been developed, but the results were evident only when administered in the early stages of the disease. It can act in three ways in these plaques: (i) by inhibiting the enzyme β-secretase with the help of BACE1 inhibitors, which decrease the production of all forms of β-amyloid, (ii) with the help of γ-secretase inhibitors (semagacestate, tarenflurbil, but the results were inadequate in phase 3 of clinical studies and avagacestate, in phase 2) and (iii) studies using α-secretase activators, such as etazolate, with good results in phase 2 of clinical studies.

As tau protein is one of the causes of Alzheimer’s disease, drugs that reduce this protein are in development. Many tau vaccines are safe and effective in laboratory animals, and a recent study has shown a good safety profile of a human anti-tau drug. Additionally, this drug even showed stimulation of the immune response [4]. As the glycation process takes place in the early stages of the disease, inhibitors of advanced glycation end products can be promising therapeutic targets for Alzheimer’s disease, such compounds being penicillamine, tenilsetam, L-carnosine, pyridoxamine, acetylsalicylic acid, etc. [5]. Gamma oscillation, a high frequency that characterizes the rhythms of brain connections, is related to interneuronal communication and can help differentiate true memories from false ones. Recently, it has been shown that the induction of these gamma oscillations has led to a decrease in the positioning of β-amyloid plaques and cognitive improvements in Alzheimer’s mice. The method is in early human studies and uses both visual and auditory stimulation [4]. Calcium channel blockers (nimodipine flunarizine, verapamil, tetrandrine) could be new treatment solutions for this disease. For example, nimodipine, a highly lipophilic L-type antagonist of calcium channels (easily crosses the blood–brain barrier), blocks calcium channels and increases cerebral blood flow. Treatment with antioxidants such as vitamin E, can decrease the rate of cell death caused by β-amyloid plaques, can alleviate neuronal toxicity and could be used in this disease. Lipid-lowering agents, isomers of ApoE protein, which may reduce the formation of β-amyloid peptides, are another alternative, as are Fe^3+^ chelators.

Immunotherapy may also be one of the most promising methods of preventing the aggregation of β-amyloid peptides. One strategy for the treatment of this disease is to modulate the activity of γ-secretase to control the β-amyloid protein ratio without affecting other activities of this enzyme complex. Therefore, γ-secretase modulators must be sufficiently selective to avoid interactions with other enzymes. Interestingly, this modulation capacity has been observed in the case of nonsteroidal anti-inflammatory drugs (NSAIDs), but most of them require toxic concentrations to be effective [6]. Carprofen is a nonsteroidal anti-inflammatory drug (NSAID) derived from propionic acid, with anti-inflammatory, analgesic and antipyretic action, today used exclusively in veterinary medicine. It inhibits the activity of cyclooxygenases 1 and 2, resulting in decreased formation of prostaglandin and thromboxane precursors. Inhibition of prostaglandin formation (through the action of prostaglandin synthetase) leads to decreased pain, inflammation and fever; inhibition of thromboxane formation has consequences in reducing platelet aggregation. The literature presents studies on carbazole derivatives with neuroprotective potential. Thus, (*E*)-3-ethyl-2-(2-(9-ethyl-9*H*-carbazol-3-yl)vinyl)benzo[d]-3-thiazole iodide or 9-(2-(2-)methoxyethoxy)ethyl)-9*H*-carbazole were synthesized and tested for the effect of inhibiting amyloid plaque formation in Alzheimer’s disease [7]. Wen et al. synthesized a number of N-substituted carbazole compounds to observe the biological protective effect of HT22 neuronal cells attacked by neurotoxins such as the neurotransmitter glutamate and homocysteic acid. Thus, 2-phenyl-9-para-tolyl-9*H*-carbazole may be a prototype molecule for the design of new compounds with activity against species associated with oxidative stress in neurodegenerative diseases [8]. In another study, conducted by Wang et al., 9-(2-chlorobenzyl)-9*H*-carbazole-3-carbaldehyde was synthesized, and they demonstrated an inhibitory effect in the production of nitrogen monoxide (NO), which is known to be involved in the aetiology of neurodegenerative diseases, along with other reactive oxygen species [9]. Schiff bases contain the azomethine group (C=N) as pharmacophore useful in the design and discovery of pharmacologically active compounds. The properties of these compounds result in potential applications in medicine. Compared to ordinary Schiff bases, hydrazonic N-acyl compounds are more stable and have a stronger biological activity because oxygen and nitrogen atoms could be involved in the formation of hydrogen bonds in biochemical systems [10]. Through structural modulations of the carprofen molecule, we obtained its derivatives, N-acyl hydrazones, which contain the azomethine (C=N) functional group of Schiff bases, aiming to study their potential in Alzheimer’s disease.

## 2. Materials and Methods

All reagents were used as received from Merck or Aldrich (Darmstadt Steinheim, Germany). The microwave-assisted synthesis was performed with a Biotage^®^ Initiator Classic 2.0 (Biotage, Uppsala, Sweden). Melting points were determined by Electrothermal 9100 apparatus (Bibby Scientific Ltd., Stone, UK) and were uncorrected. The IR spectra were recorded on a Bruker Vertex 70 FT-IR spectrometer (Bruker Corporation, Billerica, MA, USA). These were obtained using the ATR technique and are presented as w—weak band; m—medium band; s—intense band; vs—very intense band.

The ^1^H-NMR and ^13^C-NMR spectra were recorded in deuterated dimethyl sulfoxide (dmso-d6) on a Bruker Fourier 300 MHz instrument (Bruker Corporation, Billerica, MA, USA), operating at 300 MHz for ^1^H-NMR and at 75 MHz for ^13^C-NMR and on a Bruker Avance III 500 MHz instrument (Bruker Corporation, Billerica, MA, USA) operating at 500 MHz for proton and 125 MHz for carbon. In NMR spectra, chemical shifts were recorded as δ values, in parts per million (ppm), relative to tetramethyl silane as internal standard, and coupling constants (J) in Hertz. Standard abbreviations that indicate the multiplicity of signals are used as follows: s (singlet), d (doublet), t (triplet), q (quartet), m (multiplet), dd (double doublet), bs (broad singlet) and bd (broad doublet). The ^1^H- NMR data are reported in the following order: chemical shifts, multiplicity, signal/atom attribution and the coupling constants. For ^13^C NMR data, the order is as follows: chemical shifts and signal/atom attribution.

### 2.1. Chemistry

The syntheses of compounds 3 and 4 have been presented previously [10].

#### 2.1.1. General Procedure for the Synthesis of (*EZ*)-N′-Benzylidene-(*2RS*)-2-(6-chloro-9*H*-carbazol-2-yl)propanehydrazide Derivatives

A mixture of (2*RS*)-2-(6-chloro-9*H*-carbazol-2-yl)propanehydrazide (6) (Mr 287,737; 0.001 mol) and various aromatic aldehydes (0.001 mol) was introduced into a microwave tube using 3 mL absolute methanol as the reaction medium, then a few drops of glacial acetic acid were added as a catalyst. The tube was then sealed, and the samples were microwave irradiated. The method involved pre-stirring for 5 min, then irradiation for 25 min at 90 °C, at very high absorption level. The reaction mixture was cooled to room temperature and then refrigerated overnight. The product obtained was filtered and recrystallized from a mixture of isopropanol: water 1:2.

The new compounds are soluble at room temperature in DMSO, DMF and pyridine, in hot acetone, chloroform, benzene, toluene, xylene, ethyl acetate, 1,4-dioxane and lower alcohols, insoluble in water, methylene chloride and hexane.

#### 2.1.2. Spectral Data

*(EZ)-N′-(2-hydroxybenzylidene-(2RS)-2-(6-chloro-9H-carbazol-2-yl)propanehydrazide* (**1a**) (Mr = 391.85 g/mol; m.p.192–193.4 °C; yield 87%)

^1^H-NMR (300 MHz, DMSO-*d*_6_, δ ppm, J Hz): 11.82, 11.38, 11.35, 11.26 (4s, 1H, N-N-H^E/Z^); 11.16, 11.11 (2s, H-9^E/Z^); 10.07 (bs, 1H, OH); 8.99, 8.38 (2s, 1H, H-13^E/Z^); 8.15, 8.12 (2d, 1H, H-5^E/Z^, 2.2); 8.09, 8.05 (2d, 1H, H-4^E/Z^, 8.1); 7.49 (bs, 1H, H-1); 7.46–7.42 (m, 1H, H-8); 7.38–7.31 (m, 1H, H-7); 7.20–7.15 (m, 1H, H-3); 7.69–7.63 (m, 1H, H-19); 7.28–7.23 (m, 1H, H-17); 6.99–6.94 (m, 1H, H-18); 6.90–6.84 (m, 1H, H-16); 4.71, 3.85 (2q, 1H, H-10^E/Z^, 7.0); 1.49, 1.45 (2d, 3H, H-11^E/Z^, 7.0) (Figure S1).

^13^C-NMR (75 MHz, DMSO-*d*_6_, δ ppm): 174.75, 169.85 (C-12^E/Z^); 158.78, 157.27 (C-15^E/Z^); 118.74, 118.31 (C-14^E/Z^); 140.71, 140.61 (C-1a*^E/Z^*); 140.00 (C-8a); 138.50, 138.41 (C-2*^E/Z^*); 123.73 (C-5a); 123.05 (C-6); 122.99 (C-4a); 163.01, 147.26 (C-13^E/Z^); 131.35 (C-17); 130.95 (C-19); 116.59 (C-18); 116.38, 116.14 (C-16^E/Z^); 125.31, 125.21 (C-7^E/Z^); 120.65 (C-4); 119.82 (C-5); 118.98 (C-3); 112.53, 112.46 (C-8^E/Z^); 109.84, 109.79 (C-1^E/Z^); 44.46, 41.52 (C-10^E/Z^); 19.27, 18.97 (C-11^E/Z^) (Figure S2).

FT-IR (ATR in solid, ν cm^−1^): 3340m; 3175w; 3035m; 2905w; 1653vs; 1612vs; 1564s; 1463s; 1355m; 1265s; 1209s; 1152w; 1115w; 1065w; 955m; 878w; 791m; 749s; 729m; 680m; 584m.

*(EZ)-N′-(4-chlorobenzylidene-(2RS)-2-(6-chloro-9H-carbazol-2-yl)propanehydrazide* (**1b**) (Mr = 410.30 g/mol; m.p. 216–218 °C; yield 83%)

^1^H-NMR (300 MHz, DMSO-*d*_6_, δ ppm, J Hz):11.66, 11.38, 11.35, 11.33, 11.32 (5s, 2H, N-H-9, N-N-H); 8.17, 7.88 (2s, 1H, H-13^E/Z^); 8.15–8.02 (m, 2H, H-4, H-5); 7.70–7.65 (d, 2H, H-15, H-19); 7.50–7.44 (m, 4H, H-1, H-8, H-16, H-18); 7.37–7.31 (m, 1H, H-7); 7.18, 7.14 (bd, 1H, H-3^E/Z^, 7.9); 4.79, 3.85 (2q, 1H, H-10^E/Z^, 6.9); 1.48, 1.46 (2d, 3H, H-11^E/Z^, 7.0) (Figure S3).

^13^C-NMR (75 MHz, DMSO-*d*_6_, δ ppm): 174.41, 173.20, 170.31 (C-12^E/Z^); 140.70, 140.64 (C-1a^E/Z^); 140.13 (C-8a); 138.46, 138.41 (C-2^E/Z^); 134.59, 134.25 (C-14^E/Z^); 133.40, 133.31 (C-17^E/Z^); 123.78 (C-5a); 123.74 (C-6); 123.04, 122.98 (C-4a^E/Z^); 145.53, 141.52 (C-13^E/Z^); 129.08, 129.03 (C-16, C-18^E/Z^); 128.79, 128.68 (C-15, C-19^E/Z^); 125.29, 125.18 (C-7^E/Z^); 120.71, 120.49 (C-4^E/Z^); 119.79, 119.74 (C-5^E/Z^); 119.24, 119.01 (C-3^E/Z^); 112.53, 112.49 (C-8^E/Z^); 110.04, 109.80 (C-1^E/Z^); 44.46, 41.52 (C-10^E/Z^); 19.27, 18.97 (C-11^E/Z^) (Figure S4).

FT-IR(ATR in solid, ν cm^−1^): 3243s; 3228sh; 3049w; 2976w; 1643vs; 1546m; 1461m; 1344w; 1246m; 1292m; 1088m; 1007w; 930w; 858w; 816m; 737w; 604w.

*(EZ)-N′-(2-hydroxy-3-methoxy-benzylidene-(2RS)-2-(6-chloro-9H-carbazol-2-yl)propanehydrazide* (**1c**) (Mr = 421.88 g/mol; m.p. 231–232.9 °C; yield 84%)

^1^H-NMR (300 MHz, DMSO-*d*_6_, δ ppm, J Hz): 11.79, 11.39, 11.35, 11.27, 10.79, 9.42, 8.99 (7s, 3H, N-N-H^E/Z^, NH-9, OH); 8.39, 8.23 (2s, 1H, H-13^E/Z^); 8.15, 8.12 (2d, H-5^E/Z^, 2.2); 8.08, 8.05 (2d, H-4^E/Z^, 8.1); 7.49 (bs, 1H, H-1); 7.46–7.43 (m, 1H, H-8); 7.37–7.25 (m, 1H, H-7); 7.20–7.15 (m, 1H, H-3); 7.13–7.05 (m, 1H, H-19); 7.00–6.93 (m, 1H, H-18); 6.81–6.78 (m, 1H, H-17); 4.72, 3.85 (2q, H-10^E/Z^, 6.9); 3.82, 3.78 (2s, 3H, CH3O^E/Z^); 1.49, 1.45 (d, 3H, H-11^E/Z^, 6.9) (Figure S5).

^13^C-NMR (75 MHz, DMSO-*d*_6_, δ ppm): 175.95, 169.94 (C-12^E/Z^); 148.78 (C-16); 147.07 (C-15); 148.01 (C-14); 140.70 (C-1a); 140.06 (C-8a); 138.49, 138.41 (C-2^E/Z^); 123.72 (C-5a); 123.04 (C-4a); 120.64 (C-6); 147.14, 140.88 (C-13^E/Z^); 125.31, 125.21 (C-7^E/Z^); 120.76 (C-19); 120.64 (C-4); 119.80 (C-5); 119.21 (C-17); 118.96 (C-3); 113.81 (C-18); 112.54, 112.46 (C-8^E/Z^); 109.83, 109.79 (C-1^E/Z^); 56.03; 55.91 (CH3O^E/Z^); 44.65 (C-10^Z^); 43.85 (C-10^E^); 19.26 (C-11^Z^); 18.99 (C-11^E^) (Figure S6).

FT-IR(ATR in solid, ν cm^−1^): 3274vs; 3072w; 3007w; 2971w; 2935w; 2885w; 1684s; 1578m; 1464s; 1349m; 1255vs; 1191m; 1072m; 1008w; 935w; 870w; 788w; 731s; 598m.

*(EZ)-N′-(2-hydroxy-5-methoxy-benzylidene-(2RS)-2-(6-chloro-9H-carbazol-2-yl)propanehydrazide* (**1d**) (Mr = 421.88 g/mol; m.p. 215–229 °C; yield 88%)

^1^H-NMR (500 MHz, DMSO-*d*_6_, δ ppm, J Hz): 11.77, 11.32 (2s, H-9^E/Z^); 11.38, 11.28 (2s, HN^E/Z^); 10.50, 9.55 (2s, HO^E/Z^); 8.37, 8.08 (2s, H-13^E/Z^); 8.17, 8.14 (2d, H-5^E/Z^, 1.8); 8.09, 8.06 (2d, H-4^E/Z^, 8.1); 7.49, 7.41 (bs, H-1^E/Z^); 7.48, 7.45 (2d, H-8^E/Z^, 8.4); 7.35, 7.3 3(2dd, H-7^E/Z^, 1.8, 8.4); 7.19 (bd, H-3, 8.1); 7.17, 7.06 (2d, H-19^E/Z^, 2.9); 6.87, 6.78 (2dd, H-17^E/Z^, 2.9, 8.9); 6.82 (d, H-16, 8.9); 4.73, 3.85 (2q, H-10^E/Z^, 7.0); 3.75, 3.69 (2s, CH3O^E/Z^); 1.49, 1.44 (d, H-11^E/Z^, 7.0) (Figure S7).

^13^C-NMR (125 MHz, DMSO-*d*_6_, δ ppm): 174.80, 174.69, 169.76, 169.66 (C-12^E/Z^); 152.26, 152.24, 152.13, 152.10 (C-15^E/Z^); 151.29, 150.09, 150.42, 150.26 (C-18^E/Z^); 146.33, 146.24, 140.09, 140.03 (C-13^E/Z^); 140.81, 139.92 (C-8a^E/Z^); 140.64, 140.59, 140.49, 140.44 (C-2^E/Z^); 138.37, 138.30, 138.22, 138.15 (C-1a^E/Z^); 123.63, 123.58 (C-5a^E/Z^); 122.89, 122.84 (C-4a^E/Z^); 120.50, 120.46, 120.39, 120.32 (C-14^E/Z^); 120.22, 120.19 (C-6^E/Z^); 125.14, 125.06 (C-7^E/Z^); 120.68, 120.59 (C-4^E/Z^); 119.73, 119.65 (C-5^E/Z^); 119.57, 118.81 (C-3^E/Z^); 112.38, 112.32 (C-8^E/Z^); 109.66, 109.60, 109.40, 109.34 (C-1^E/Z^); 118.08, 117.96 (C-17^E/Z^); 117.19, 117.09 (C-16^E/Z^); 112.09, 112.04, 109.13, 109.09 (C-19^E/Z^); 55.48, 55.40 (CH3O^E/Z^); 44.36, 44.33, 41.59, 41.55 (C-10^E/Z^); 19.33, 18.90 (C-11^E/Z^) (Figure S8).

FT-IR(ATR in solid, ν cm^−1^): 3388s; 3345m; 3004w; 2969w; 1687vs; 1620m; 1580w; 1525m; 1489s; 1453vs; 1385w; 1317m; 1263s; 1189s; 1160s; 1063w; 1027m; 958w; 942w; 873w; 829m; 802m; 782m; 693w.

*(EZ)-N′-(2,6-dichloro-benzylidene-(2RS)-2-(6-chloro-9H-carbazol-2-yl)propanehydrazide* (**1e**) (Mr = 444.74 g/mol; m.p. 242–251 °C; yield 74.2%)

^1^H-NMR (500 MHz, DMSO-*d*_6_, δ ppm, J Hz): 11.82, 11.58 (2s, H-9^E/Z^); 11.40, 11.32 (2s, HN^E/Z^); 8.40, 8.23 (2s, H-13^E/Z^); 8.17, 8.13 (2d, H-5^E/Z^, 1.6); 8.14, 8.04 (2d, H-4^E/Z^, 8.1); 7.54 (d, 2H, H-16, H-18^E/Z^, 8.1); 7.40 (t, 1H, H-17^E/Z^, 8.1); 7.52–7.44 (m, 2H, H-1, H-8); 7.33–7.37 (m, H-7^E/Z^); 7.20, 7.14 (2dd, H-3^E/Z^, 1.1, 8.1); 4.78, 3.86 (2q, H-10^E/Z^, 7.0); 1.49, 1.46 (2d, H-11^E/Z^, 7.0) (Figure S9).

^13^C-NMR (125 MHz, DMSO-*d*_6_, δ ppm): 175.56, 175.45, 170.10, 170.02 (C-12^E/Z^); 142.00, 138.05 (C-13^E/Z^); 140.57, 140.42 (C-8a^E/Z^); 140.10, 139.90 (C-1a^E/Z^); 138.30, 138.15 (C-2^E/Z^); 133.88, 133.85 (C-15, C-19^E/Z^); 130.53, 129.68 (C-14^E/Z^); 123.62, 123.58 (C-5a^E/Z^); 122.90, 122.83 (C-4a^E/Z^); 120.27, 120.23 (C-6^E/Z^); 131.14, 130.95 (C-17^E/Z^); 129.42, 129.03 (C-16, C-18^E/Z^); 125.16, 125.02 (C-7^E/Z^); 120.74, 120.43 (C-4^E/Z^); 119.65, 119.57 (C-3^E/Z^); 119.08, 118.81 (C-5^E/Z^); 112.32, 112.27 (C-8^E/Z^); 109.88, 109.82, 109.73, 109.67 (C-1^E/Z^); 44.58, 44.55, 40.73, 40.70 (C-10^E/Z^); 19.11, 19.04 (C-11^E/Z^) (Figure S10).

FT-IR(ATR in solid, ν cm^−1^): 3444m; 3198w; 3050w; 2978w; 2935w; 1657vs; 1550vs; 1467m; 1429m; 1364w; 1311w; 1270w; 1235w; 1192s; 1069m; 961w; 881w; 801w; 775m; 724w; 694w.

*(EZ)-N′-(3,5-dichloro-benzylidene-(2RS)-2-(6-chloro-9H-carbazol-2-yl)propanehydrazide* (**1f**) (Mr = 444.74 g/mol; m.p. 239–244 °C; yield 76.4%)

^1^H-NMR (300 MHz, DMSO-*d*_6_, δ ppm, J Hz): 11.82, 11.52 (2s, H-9^E/Z^); 11.38, 11.31 (2s, HN^E/Z^); 8.12, 7.86 (2s, H-13^E/Z^); 8.17, 8.14 (2d, H-5^E/Z^, 1.9); 8.10, 8.06 (2d, H-4^E/Z^, 8.2); 7.69, 7.66 (2d, H-15, H-19^E/Z^, 1.9); 7.62, 7.59 (t, H-17^E/Z^, 1.9); 7.50–7.40 (m, 2H, H-1, H-8); 7.33–7.37 (m, H-7); 7.19 (dd, H-3, 1.1, 8.2); 4.81, 3.88 (2q, H-10^E/Z^, 7.0); 1.48, 1.46 (2d, H-11^E/Z^, 7.0) (Figure S11).

^13^C-NMR (75 MHz, DMSO-*d*_6_, δ ppm): 175.47, 175.37, 170.27, 170.17 (C-12^E/Z^); 143.40, 139.49 (C-13^E/Z^); 140.65, 140.55 (C-8a^E/Z^); 139.86 (C-1a); 138.34, 138.28, 138.20, 138.14 (C-2^E/Z^); 138.04 (C-14); 134.56, 134.52 (C-16, C-18^E/Z^); 123.58 (C-5a); 122.86, 122.80 (C-4a^E/Z^); 120.47, 120.20 (C-6^E/Z^); 128.88, 128.60 (C-17^E/Z^); 125.21, 125.11 (C-15, C-19^E/Z^); 125.03 (C-7); 120.61, 120.47 (C-4^E/Z^); 119.66, 119.58 (C-5^E/Z^); 119.10, 118.76 (C-3^E/Z^); 112.35, 112.29 (C-8^E/Z^); 109.65, 109.60 (C-1^E/Z^); 44.44, 41.34 (C-10^E/Z^); 19.07, 18.82 (C-11^E/Z^) (Figure S12).

FT-IR (ATR in solid, ν cm^−1^): 3326m; 3195w; 3040w; 2906w; 1639s; 1566s; 1454m; 1352w; 1272w; 1243m; 1203m; 1098w; 1070w; 1015w; 954w; 921w; 859w; 810m; 733w; 672w.

### 2.2. Bioinformatic Procedure

#### 2.2.1. Molecular Modelling of (*EZ*)-N′-Benzylidene-(*2RS*)-2-(6-chloro-9*H*-carbazol-2-yl)propanehydrazide Derivatives and Drug-Like Character Evaluation

The chemical structures (*EZ*)-N′-benzylidene-(2*RS*)-2-(6-chloro-9*H*-carbazol-2-yl)propanehydrazide derivatives were built by Expasy portal [11] with obtaining of SMILES files. Predicted drug-like and bioavailability features of compounds were evaluated by Lipinski, Ghose, Egan and Veber rules [12]. The compounds SMILES file was loaded into the ExPASy Bioinformatics Resource Portal/ SwissADME [13].

#### 2.2.2. Computational Pharmacokinetics and Pharmacogenomics Profiles of (*EZ*)-N′-Benzylidene-(*2RS*)-2-(6-chloro-9*H*-carbazol-2-yl)propanehydrazide Derivatives

For the assessment of ADME-Tox (Absorption, Distribution, Metabolism, Excretion and Toxicity) features, the SMILES files of **1a**–**f** compounds were loaded in pkCSM database [14]. In our study, we quantitatively and qualitatively evaluated an important number of ADME-Tox features, but here we present the most significant features such as (1) Absorption—(i) human intestinal absorption (in %), a molecule with an absorbance of less than 30% is considered to be poorly absorbed; (2) Distribution—(i) blood–brain barrier (BBB) permeability (Numeric (log BBB)), if log BBB > 0.3, that means the tested compounds have good BBB permeability, and if log BBB < −1, they have low BBB permeability; (ii) central nervous system (CNS) permeability (Numeric (log Ps)), if log Ps > −0.2, the compounds would penetrate CNS, and if log Ps < −3, they would not enter CNS; (3) Excretion—feature of renal organic cation transporter 2 (OCT2), substrate OCT2.

The pharmacogenomic profile of compounds was represented by our evaluation of inhibitor or substrate profiles of **1a**–**f** compounds a few very important cytochromes in neuropsychiatric metabolization drugs, namely, CYP2D6, CYP3A4, CYP1A2, CYP2C19 and CYP2C9. An important part of our study was represented by the prediction of the toxicity. Using pkCSM webservers, we evaluated the AMES toxicity, hepatotoxicity, LD50 (median lethal dose) and Max. tolerated dose (human) [14].

#### 2.2.3. Computational Pharmacodynamic Profiles of (*EZ*)-N′-Benzylidene-(*2RS*)-2-(6-chloro-9*H*-carbazol-2-yl)propanehydrazide Derivatives and Their Predicted ICD-10 Association

The SwissTargetPrediction database allows us to predict the targets of a small molecule, using molecular similarities, comparing the molecule with the database containing 280.000 active compounds, with more than 2000 targets, from 5 different organisms. Supplementing this was the bioinformatics HitPick: Hit Identification & Target Prediction [15]. The different output files contain specific targets and the probability of compounds to be a ligand for those specific targets.

By using the PROMISCUOUS2 [16] drug-repositioning tool using the machine learning models and SMILES files of compounds **1a**–**f**, we predicted all ICD-10 (International Statistical Classification of Diseases and Related Health Problems 10th Revision) categories associated.

## 3. Results

### 3.1. Chemistry

For the synthesis of the (*EZ*)-N′-benzylidene-(2*RS*)-2-(6-chloro-9*H*-carbazol-2-yl)propanehydrazide derivatives (**1a**–**f**), we used (*RS*)-2-(6-chloro-9*H*-carbazol-2-yl)propanoic acid (carprofen) (**2**), which was converted, in the presence of concentrated sulfuric acid and absolute excess methanol, to the corresponding methyl ester (**3**). This gave (2*RS*)-2-(6-chloro-9*H*-carbazol-2-yl)propanohydrazide (carprofen hydrazide) (**4**), by refluxing in ethyl alcohol with 100% hydrazine hydrate. The last stage of preparation of the derivatives (**1a**–**f**) consisted of the reaction of the hydrazide of carprofen (**4**) with aromatic aldehydes, in absolute methanol, by microwave irradiation.

**Scheme 1:**
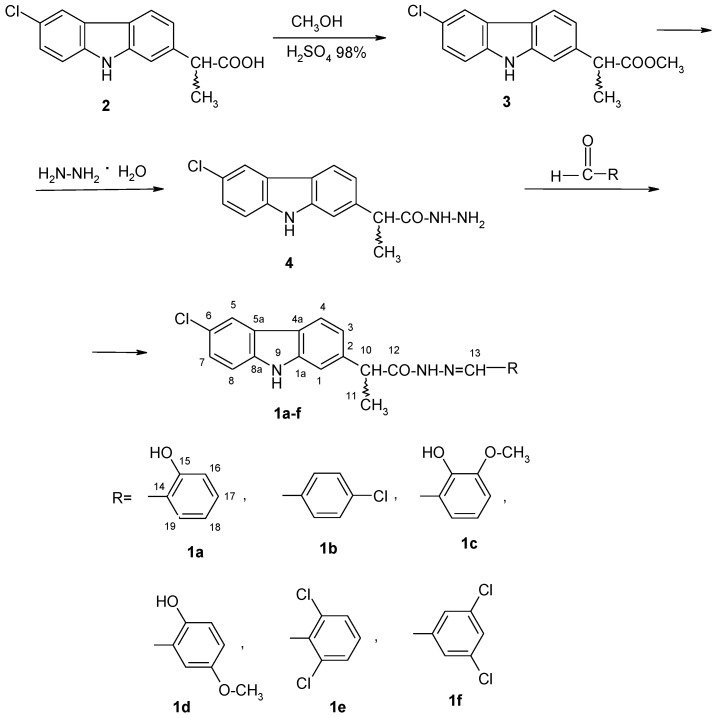
Synthesis scheme of new (*EZ*)-N′-benzylidene-(2*RS*)-2-(6-chloro-9*H*-carbazol-2-yl)propanehydrazide derivatives (**1a**–**f**).

The method of synthesis of the new compounds is presented in Scheme 1.

### 3.2. Bioinformatics

#### 3.2.1. Drug Likeness Evaluation of (*EZ*)-N′-Benzylidene-(*2RS*)-2-(6-chloro-9*H*-carbazol-2-yl)propanohydrazide Derivatives

By calculating the molecular descriptors using Lipinski, Veber, Ghose, Egan rules, we noticed that compounds **1a**, **c**, **d** abide simultaneously all medicinal chemistry rules and have a good bioavailability score, according to Table 1.

Instead, compounds **1b**, **1e** and **1f** do not follow the Ghose and Egan rules regarding hydrophobicity (Table 1). Related to our results, we can mention that de novo compounds **1a**–**f** proposed here may present potential drug features and can be considered in further pharmacokinetic and pharmacodynamic studies.

#### 3.2.2. Computational Pharmacokinetics and Pharmacogenomics Profiles of (*EZ*)-N′-Benzylidene-(*2RS*)-2-(6-chloro-9*H*-carbazol-2-yl)propanohydrazide Derivatives

To identify critical absorption and distribution features of de novo compounds, we computed the intestinal absorption, blood–brain barrier permeability as well as central nervous system permeability (Table 2) [14,17].

Organic Cation Transporter 2 (OCT2) is a renal uptake transporter that participates in the first step of active renal secretion of cationic drugs [18]. Evaluation of a compound interaction with OCT2 is recommended by both European Medicines Agency (EMA) and Food and Drug Administration (FDA) guidelines [19]. By predicting the OCT2 renal substrate, we noticed that all compounds involved in the study do not present OCT2 affinities (Table 2).

In Table 3, we present whether compounds **1a**–**f** are inhibitors or substrate (are metabolized) for cytochromes family P450 (CYP) with emphasis on (i) CYP2D6 (involved in the metabolism of ~25% of the drugs [20], (ii) CYP3A4 (involved in the metabolism of up to 50% of existent drugs [21] (iii) CYP1A2 (involved in the metabolism of 10% of existing drugs), (iv) CYP2C19 (participate in the metabolism of benzodiazepines, antidepressants, etc.), (v) CYP2C9 (involved in the metabolism of ~15% of the drugs that are CYP metabolized). CYP enzymes polymorphisms may result in potentiated, reduced or even total loss of function of CYP activity [22]. If a drug that is metabolised by a CYP enzyme is Co-administrated with an inhibitor of the same CYP may lead to a rise in the drug level (of the drug that is metabolised by that CYP). Concerning the previous sentence is important to predict the interaction of our compounds with most commune CYP enzymes (Table 3).

#### 3.2.3. Computational Pharmacodynamic Profiles of (*EZ*)-N′-Benzylidene-(*2RS*)-2-(6-chloro-9*H*-carbazol-2-yl)propanehydrazide Derivatives and Their Predicted ICD-10 Association

An essential aim of our study was represented by the prediction of the pharmacodynamic features of compounds by accessing the Swiss Target Prediction database [23] and HitPick database [15]. With these tools, we estimated the most probable macromolecular targets of small molecules.

Considering the literature data, we take into consideration the microtubule-associated protein tau database [23] and PTG S1,S2 [15] (Table 4). Our predicted results are presented in Table 4.

Another aim in our work is to predict the clinical applicability of compounds **1a**–**f** in neuropsychiatric disorders by searching for similarity among these compounds and clinical used drugs applying advanced machine learning [16]. From ICD-10, we selected the just items specific for brain disorders such as mental and behavioural disorders due to psychoactive substance use, mood (affective) disorders and extrapyramidal and movement disorders (Table 4).

## 4. Discussion

Obtaining N-acyl hydrazones was performed by using a microwave-assisted synthesis method. We chose this method, comparing the main defining characteristics of chemical syntheses by conventional methods, as well as those of microwave irradiation. The presence in the molecule of the amide and imine groups leads to the appearance of stereoisomers (E, Z) and sin/anti periplanar conformations due to the CONH group. Structural characterization by analysis of ^1^H-NMR spectra confirms multiple signals due to stereoisomers or conformers in the mixture. Moreover, due to the carbon atom bound to the CO group, which is chiral, there are two stereoisomers, R and S, in equal proportions, which can attract a lot of NMR signals, observable especially in the carbon spectra and less distinct in the spectra of hydrogen. In the case of the studied carprofen derivatives, the presence of the chiral centre does not induce additional discrimination and only the E/Z isomerism matters.

**Figure 1:**
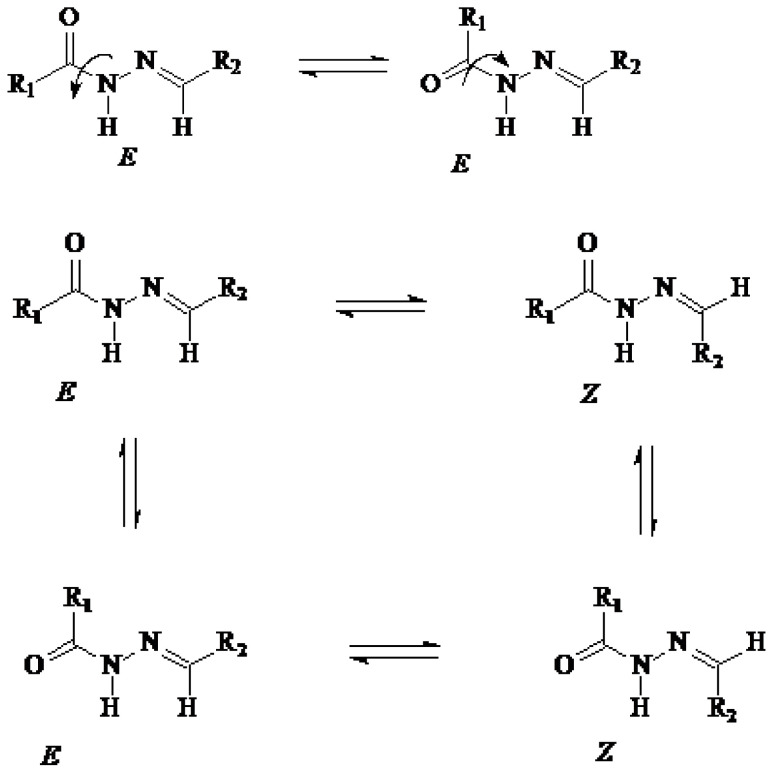
N-acyl hydrazone conformers mixture.

N-Acylhydrazones of aromatic aldehydes exist in solution as a mixture of the conformers because of the hindered rotation on the C-N amide bond (Figure 1) [24,25,26,27,28,29].

For compound 1a, the ratio between stereoisomers is 1/0.3; the more intense signal is assigned to stereoisomer E. In the case of compounds **1b** and **1c**, the ratio of stereoisomers E/Z is 1.0/0.7.

In the case of compounds **1a** and **1c**, the hydrogen bond between hydroxyl H and imine N can stabilize an antiperiplanar conformation of the E isomer.

For compound **1d**: (i) the ratio between E-R + E-S/E-R + E-S diastereomers determined from the value of ^1^H-NMR integrals on H-10 is 0.68 which indicates an excess of one of the pairs of E-Z diastereomers; (ii) by adding TFA the signals from δ = 10.50 and δ = 9.55 ppm disappear and thus confirm the assignment of HO; the other NH signals remaining unchanged; (iii) the H-13 signal from the pair of diastereoisomers E and Z (located near the double bond and the chiral center at C-10), appears in the form of several singlets (at a distance of ~2–5 Hz); in addition to the E/Z isomerism, the amide group, due to the restricted rotation, leads to the appearance of *syn-* and *anti*-periplanar conformations stabilized by hydrogen bonds; (iv) the C-10 signal appears in aldazine derivatives in the form of two signals, one major and one minor, as they belong to type E or Z diastereomers; in the carbon spectra recorded at 125 MHz, the presence of optically active C-10 is noted by a secondary splitting, due to the chiral centre or *syn*/*anti*-periplanar conformations; 44.37 (C-10 E-*syn*); 44.34 (C-10E-*anti*); 41.59 (C-10Z-*syn*); 41.56 (C-10Z-*anti*); (v) for the carbon atoms close to the aldazine double bond and to the chiral C-10, four distinct signals are observed due to the mentioned pairs of diastereoisomers. This is best seen in C-19 where the difference between Z vs. E structure is ~3 ppm.

In compound **1e**, diastereoisomers E and Z are 0.3/0.7.

For compound **1f**, the ratio of E-*syn* + E-*anti*/Z-*syn* + Z-*anti* diastereomers, determined from the value of ^1^H-NMR integrals on H-10, is 1: 1. The carbon spectrum of this compound (recorded at 75 MHz), compared to compound **1d** (which was recorded at 125 MHz) contains virtually all the signals of the diastereoisomers shown. In most signals, they are overlapping or close and cannot be individualized. For C-12, all four values are presented.

The interpretation of the FT-IR spectra allowed us to highlight the way the reactions occurred. The first elements highlighted were the absence of bands from values around 1720 and 3400 cm^−1^ due to carbonyl v(C=O) and ν(NH_2_) functional groups from starting reagents. This means that the functional groups hydrazino and aldehyde moieties of the starting reagents no longer exist and have been condensed and converted into their Schiff bases. The most important aspect was the appearance of a very strong or strong intensity absorption bands at 1639–1687 cm^−1^ assigned to azomethine (v (HC=N)) stretching vibrations. Intense or medium signals were also recorded at 1027–1069 cm^−1^ corresponding to v (N-N) bond. The C-O stretching vibrations appear at the 1240–1270 cm^−1^ range as strong or medium bands. Additionally, in the range 3201–3215 cm^−1^, we recorded the characteristic signals due to N-H group from carbazole nucleus. The aromatic nuclei give characteristic signals in the 1500–1400, 1100–1050 and 900–700 cm^−1^ region. The broad bands between 2900 and 3000 cm^−1^ in the spectra of the compounds **1a**, **1c**, and **1d** demonstrate the formation of the intramolecular hydrogen bond between the salicyl part (OH proton) and the nitrogen atom. The signals between 3000 and 2800 cm^−1^ correspond to methoxy group stretching vibrations. The C-Cl stretching vibration is seen about 600–700 cm^−1^ as weak or medium bands.

The drug-like evaluation of (*EZ*)-N′-benzylidene-(*2RS*)-2-(6-chloro-9*H*-carbazol-2-yl)propanohydrazide derivatives reveals that compounds **1b**, **1e** and **1f** had one violation of Lipinski’s rule of 5 (MILOGP is higher than 4.15). Nevertheless, Lipinski’s rule says that a compound must respect a minimum of three criteria, so our derivatives follow the Lipinsky rule [30]. The Veber rule is respected by our derivatives; therefore, all of them may have a good bioavailability when are administrated orally. Compounds **1b**, **1e** and **1f**, infringe Ghose and Egan filters (Table 1) [13,31].

ADME-Tox predictions show that those new derivatives have good intestinal absorption (under 30% is suboptimal). Due to the intention to use those compounds in brain disorders, we consider the BBB and CNS permeability of the compounds. Our predictions show that derivatives **1b**, **1e**, and **1f** present a good BBB permeability, but **1a**, **1c** and **1d** do not. The CNS permeability predictions reveal that all compounds expect to penetrate the CNS, with good CNS permeability of compounds **1e** and **1f**.

We are highly interested in studying the pharmacogenomic profiles of our compounds in interaction with the cytochrome CYP2D6, CYP3A4 and CYP2C9 family. They are strongly involved in the metabolism of neuropsychiatric drugs (Table 3).

Our results revealed that compounds **1a**–**f** are not CYP2D6 inhibitors, and just **1a** and **1f** may be CYP2D6 substrate. Instead, all compounds develop a CYP3A4 substrate and inhibitors profiles (except **1a**). Regarding CYP1A2, CYP2C19, and CYP2C9 activities profiles all of the compounds presented inhibitory actions. (Table 3)

Toxicity predictions show that (according to AMES toxicity predictions) **1a**–**f** derivatives have mutagenic potential, are hepatotoxic, and inhibitors for hERG II but not hERG I (Table 2) [14].

The **1a**–**f** Schiff bases, (*EZ*)-N′-benzylidene-(2*RS*)-2-(6-chloro-9*H*-carbazol-2-yl)propanohydrazide derivatives, presented not high similarity with the substrate of the microtubule-associated tau protein [32]). Instead, our compounds presented a suitable similarity to PTGS1 and PTGS2 substrates, indicating a possible anti-inflammatory activity [33].

By ICD-10 prediction, we noticed that compounds **1b**, **1e** and **1f**, can be used in mental and behavioural disorders, mood disorders, extrapyramidal and movement or other degenerative diseases of the nervous system (in case of 1e compound only). The predicted ICD-10 for compound **1c** reveals a possibility of using just in extrapyramidal and movement disorders (Table 4).

## 5. Conclusions

Starting from carprofen, we obtained new molecules, N-acyl hydrazones, which were characterized physiochemically and spectrally by FTIR and NMR analysis. We considered the microwave-assisted synthesis of these compounds, considering the fact that it falls among the green syntheses. By bioinformatics tools we obtained that that all compounds develop a CYP3A4 substrate and inhibitors profiles (except **1a**). All of the compounds presented inhibitory actions regarding CYP1A2, CYP2C19, and CYP2C9 activities profiles. Compounds **1b**, **1e** and **1f**, can be used in mental and behavioural disorders, mood disorders, extrapyramidal and movement or other degenerative diseases of the nervous system (in case of 1e compound only). Compounds **1e** and **1f** have also a good CNS permeability and a good BBB permeability. Compound **1e**, (*EZ*)-N′-(2,6-dichloro-benzylidene-(2*RS*)-2-(6-chloro-9*H*-carbazol-2-yl)propanohydrazide, has a high potential to be used as a good candidate in neurodegenerative disorders treatment.

## Figures and Tables

**Table 1:** The bioavailability, drug-likeness and lead-likeness features of compounds **1a**–**f** [13].

Compound	Lipinski	Ghose	Veber	Egan	Bioavailability Score
1a	yes	yes	yes	yes	0.55
1b	yes	no	yes	no	0.55
1c	yes	yes	yes	yes	0.55
1d	yes	yes	yes	yes	0.55
1e	yes	no	yes	no	0.55
1f	yes	no	yes	no	0.55

**Table 2:** ADME-Tox evaluation of **1a**–**f** compounds.

ADME-Tox Features	1a	1b	1c	1d	1e	1f	Unit
Intestinal absorption (human)	87.95	86.98	89.11	90.46	86.88	88.06	% Absorbed
BBB permeability	−0.93	0.13	−0.92	−1.12	0.07	0.12	log BB
CNS permeability	−1.69	−1.43	−1.91	−1.93	−1.33	−1.31	log PS
Renal OCT2 substrate	no	no	no	no	no	no	yes/no
AMES toxicity	yes	yes	yes	yes	yes	yes	yes/no
Max. tolerated dose (human)	0.34	0.43	0.05	0.68	0.41	0.23	log mg/kg/day
hERG I inhibitor	no	no	no	no	no	no	yes/no
hERG II inhibitor	yes	yes	yes	yes	yes	yes	yes/no
Oral Rat Acute Toxicity (LD50)	2.17	1.79	1.29	1.90	1.84	1.99	mol/kg
Hepatotoxicity	yes	yes	yes	yes	yes	yes	yes/no

**Table 3:** Pharmacogenomic profile of compounds **1a**–**f** targeting CYP2D6, CYP3A4 CYP1A2, CYP2C19 CYP2C9 [14].

P450 Cytocroms	1a	1b	1c	1d	1e	1f
CYP2D6 substrate	yes	no	no	no	no	yes
CYP3A4 substrate	yes	yes	yes	yes	yes	yes
CYP1A2 inhibitior	yes	yes	yes	yes	yes	yes
CYP2C19 inhibitior	yes	yes	yes	yes	yes	yes
CYP2C9 inhibitior	yes	yes	yes	yes	yes	yes
CYP2D6 inhibitior	no	no	no	no	no	no
CYP3A4 inhibitior	no	yes	yes	yes	yes	yes

**Table 4:** The predicted affinity of compounds **1a**–**f** at microtubule-associated protein tau, PTGS1/PTGS 2 and their predicted brain disorders applicability.

Enzyme	1a	1b	1c	1d	1e	1f
Microtubule-associated protein tausubstrate similarity	0.33	0.27	0.20	0.20	0.24	0.26
PTGS1/PTGS 2 substrate similarity	0.44	0.50	0.41	0.40	0.47	0.47
**ICD-10 substance use Probability**						
F10-F19:Mental and behavioural disorders due to psychoactive	-	0.85	-	-	0.86	0.85
F30-F39: Mood (affective) disorders	-	0.83	-	-	0.84	0.83
G20-G26: Extrapyramidal and movement disorders	-	0.80	0.81	-	0.82	0.80
G30-G32: Other degenerative diseases of the nervous system	-	-	-	-	0.83	-

## Data Availability

The data presented in this study are available on request from the corresponding author.

## Supplementary Materials

The following are available online, Figure S1: The ^1^H-NMR spectrum of (EZ)-N′-(2-hydroxybenzylidene-(2RS)-2-(6-chloro-9H-carbazol-2-yl)propanehydrazide (1a), Figure S2: The ^13^C-NMR spectrum of (EZ)-N′-(2-hydroxybenzylidene-(2RS)-2-(6-chloro-9H-carbazol-2-yl)propanehydrazide (1a); Figure S3: The ^1^H-NMR spectrum of (EZ)-N′-(4-chlorobenzylidene-(2RS)-2-(6-chloro-9H-carbazol-2-yl)propanehydrazide (1b), Figure S4: The ^13^C-NMR spectrum of (EZ)-N′-(4-chlorobenzylidene-(2RS)-2-(6-chloro-9H-carbazol-2-yl)propanehydrazide (1b), Figure S5: The ^1^H-NMR spectrum of (EZ)-N′-(2-hydroxy-3-methoxy-benzylidene-(2RS)-2-(6-chloro-9H-carbazol-2-yl)propanehydrazide (1c), Figure S6: The ^13^C-NMR spectrum of (EZ)-N′-(2-hydroxy-3-methoxy-benzylidene-(2RS)-2-(6-chloro-9H-carbazol-2-yl)propanehydrazide (1c), Figure S7: The ^1^H-NMR spectrum of (EZ)-N′-(2-hydroxy-5-methoxy-benzylidene-(2RS)-2-(6-chloro-9H-carbazol-2-yl)propanehydrazide (1d), Figure S8: The ^13^C-NMR spectrum of (EZ)-N′-(2-hydroxy-5-methoxy-benzylidene-(2RS)-2-(6-chloro-9H-carbazol-2-yl)propanehydrazide (1d), Figure S9: The ^1^H-NMR spectrum of (EZ)-N′-(2,6-dichloro-benzylidene-(2RS)-2-(6-chloro-9H-carbazol-2-yl)propanehydrazide (1e), Figure S10: The ^13^C-NMR spectrum of (EZ)-N′-(2,6-dichloro-benzylidene-(2RS)-2-(6-chloro-9H-carbazol-2-yl)propanehydrazide (1e), Figure S11: The ^1^H-NMR spectrum of (EZ)-N′-(3,5-dichloro-benzylidene-(2RS)-2-(6-chloro-9H-carbazol-2-yl)propanehydrazide (1f), Figure S12: The ^13^C-NMR spectrum of (EZ)-N′-(3,5-dichloro-benzylidene-(2RS)-2-(6-chloro-9H-carbazol-2-yl)propanehydrazide (1f).

## Sample Availability

Samples of the compounds **1a**–**f** are available from the authors.
