# Supplementary material for: Synthesis and Bioinformatic Characterization of New Schiff Bases with Possible Applicability in Brain Disorders
Source: Molecules. 2021 Jul 8;26(14):4160. doi: 10.3390/molecules26144160 (PMC8307098; doi:10.3390/molecules26144160)
Supplement: Supplementary file 1 [file molecules-26-04160-s001.zip › molecules-1269818-supplementary.pdf]

Supplementary Material

Synthesis and Bioinformatic Characterization of New Schiff Bases with Possible Applicability in Brain Disorders

Speranta Avram [1], Ana Maria Udrea [2], Diana Camelia Nuta[3], Carmen Limban[3], Adrian Cosmin Balea [3], Miron Teodor Caproiu[4], Florea Dumitrascu[4], Cătălin Buiu[5]*, Alexandra Teodora Bordei [3]

[1] Department of Anatomy, Animal Physiology, and Biophysics, Faculty of Biology, University of Bucharest, 36-46 M. Kogălniceanu Boulevard, 050107, Bucharest, Romania; speranta.avram@gmail.com

[2] National Institute of Laser, Plasma and Radiation Physics, 409 Atomistilor str., RO-077125Magurele, Ilfov, Romania; ana.udrea@inflpr.ro

[3] Department of Pharmaceutical Chemistry, Faculty of Pharmacy, "Carol Davila" University of Medicine and Pharmacy, 6 TraianVuia Street, 020956, Bucharest, Romania; carmen.limban@umfcd.ro (C.L.); diana.nuta@umfcd.ro (D.C.N.); adrian-cosmin.balea@rez.umfcd.ro (A.C.B.); alexandra.telehoiu@drd.umfcd.ro(A.T.B.)

[4] The Organic Chemistry Center of Romanian Academy “C. D. Neniţescu”, SplaiulIndependenţei 202B, Bucharest, 060023, Romania; dorucaproiu@gmail.com (M.C.); fdumitra@yahoo.com (F.D.)

[5] Department of Automatic Control and Systems Engineering, Politehnica University of Bucharest, Spl. Independenţei 313, Bucharest, 060042 Romania; catalin.buiu@upb.ro

* Correspondence: catalin.buiu@upb.ro; Tel.: 00-40-214029167

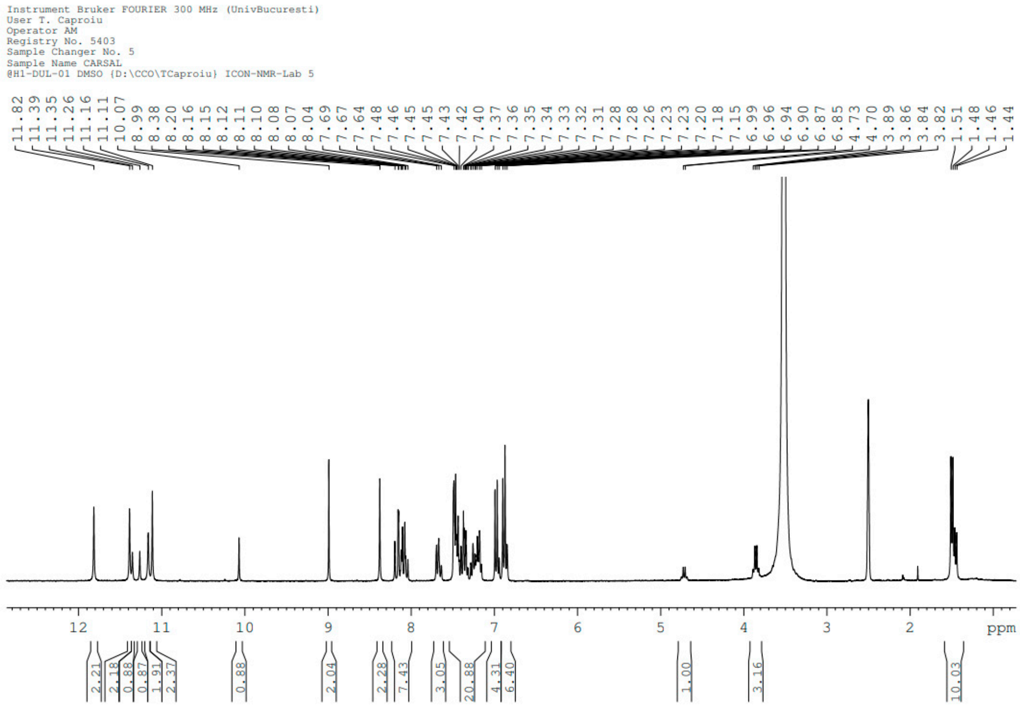

Figure S1. The ^{1}H-NMR spectrum of (*EZ*)-N'-(2-hydroxybenzylidene-(2*RS*)-2-(6-chloro-9*H*-carbazol-2-yl)propanehydrazide (**1a**)

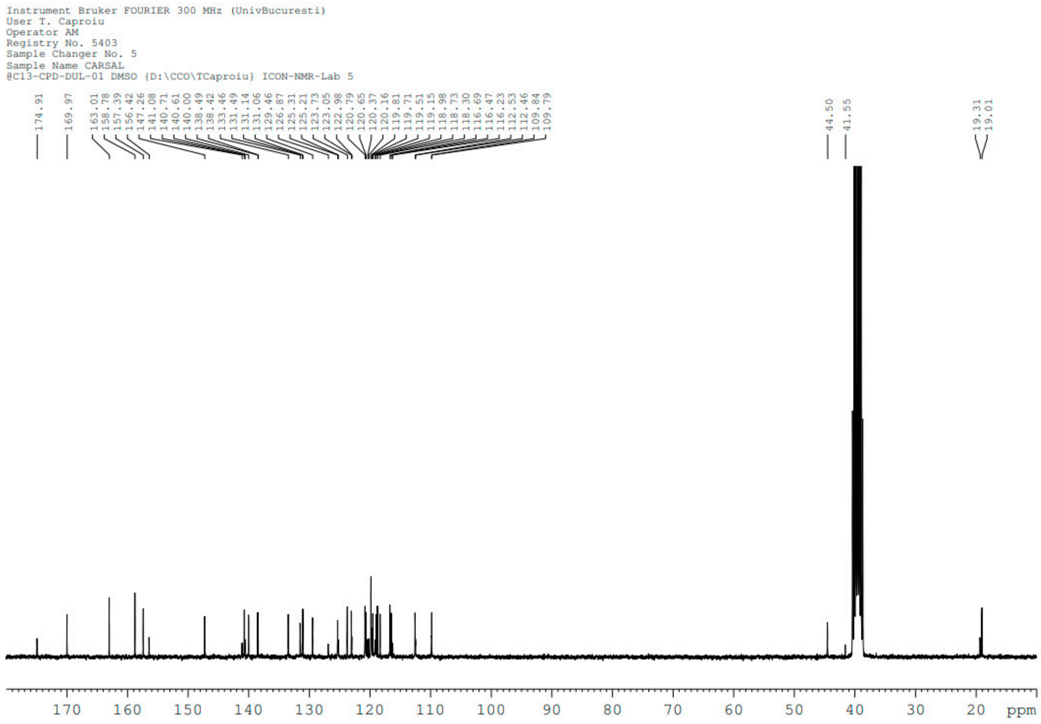

Figure S2. The ^{13}C-NMR spectrum of (*EZ*)-N'-(2-hydroxybenzylidene-(2*RS*)-2-(6-chloro-9*H*-carbazol-2-yl)propanehydrazide (**1a**)

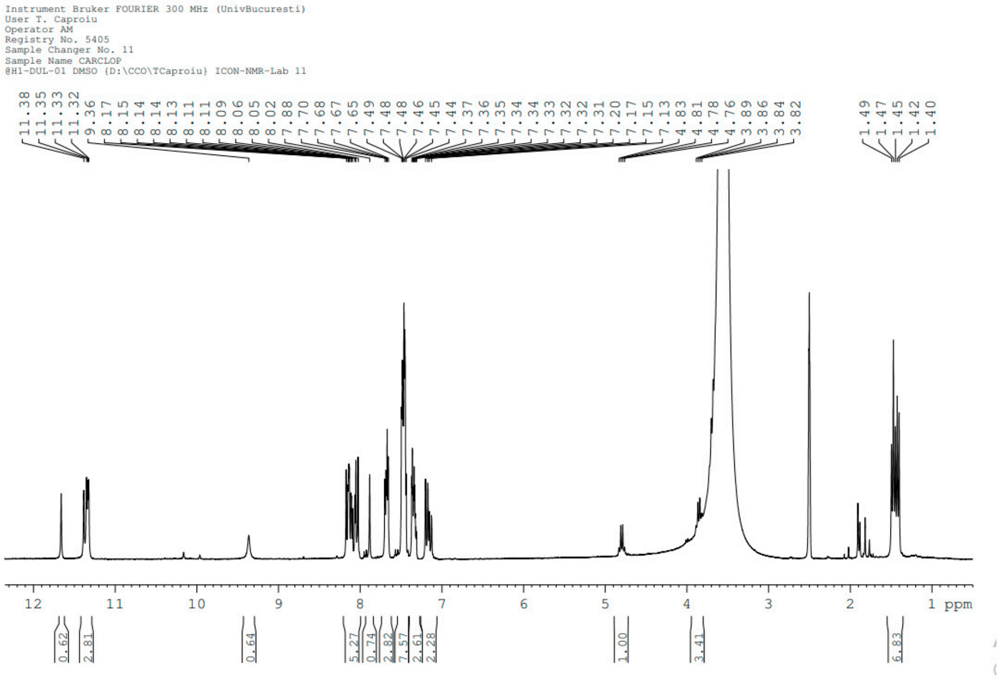

Figure S3. The ^{1}H-NMR spectrum of (*EZ*)-N'-(4-chlorobenzylidene-(2*RS*)-2-(6-chloro-9*H*-carbazol-2-yl)propanehydrazide (**1b**)

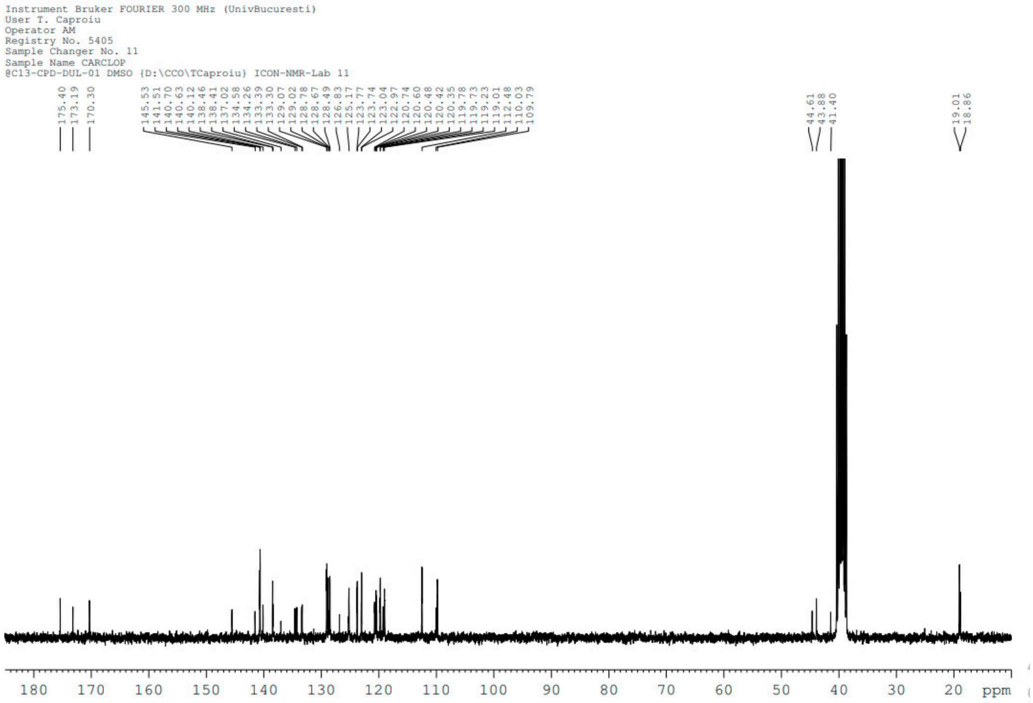

Figure S4. The ^{13}C-NMR spectrum of (*EZ*)-N'-(4-chlorobenzylidene-(2*RS*)-2-(6-chloro-9*H*-carbazol-2-yl)propanehydrazide (**1b**)

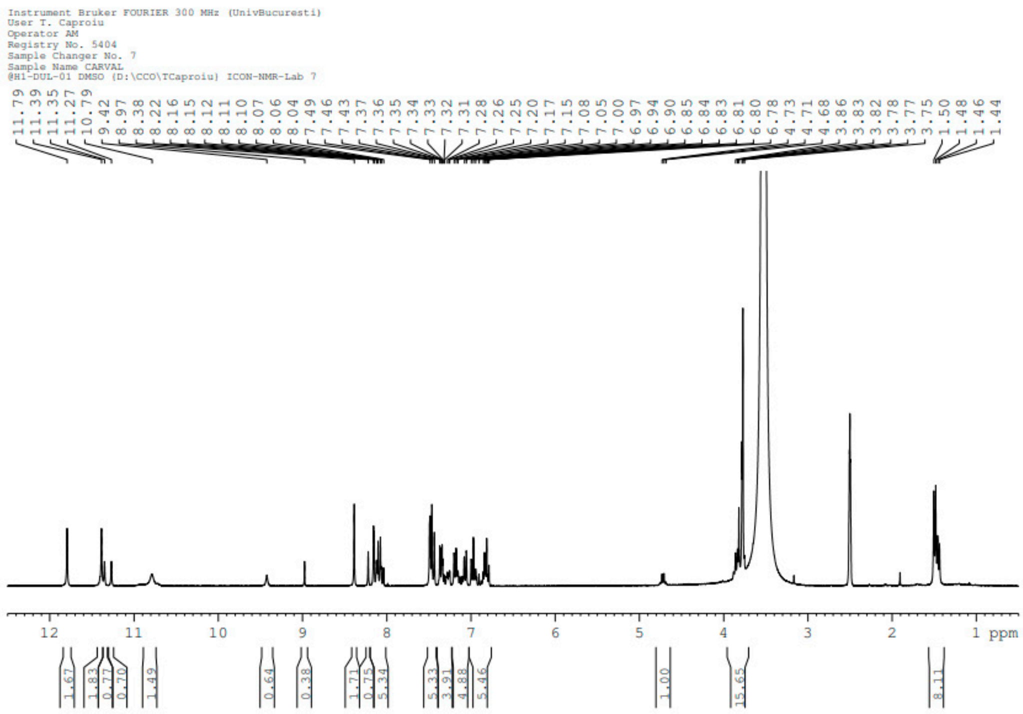

Figure S5. The ^{1}H-NMR spectrum of (*EZ*)-N'-(2-hydroxy-3-methoxy-benzylidene-(2*RS*)-2-(6-chloro-9*H*-carbazol-2-yl)propanehydrazide (**1c**)

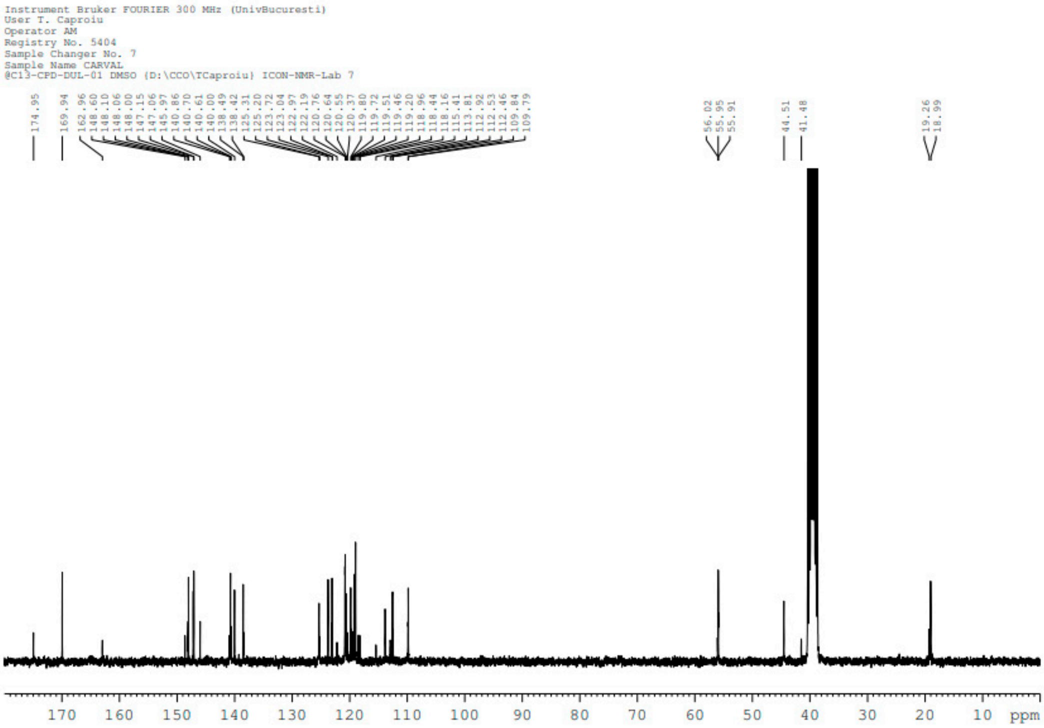

Figure S6. The ^{13}C-NMR spectrum of (*EZ*)-N'-(2-hydroxy-3-methoxy-benzylidene-(2*RS*)-2-(6-chloro-9*H*-carbazol-2-yl)propanehydrazide (**1c**)

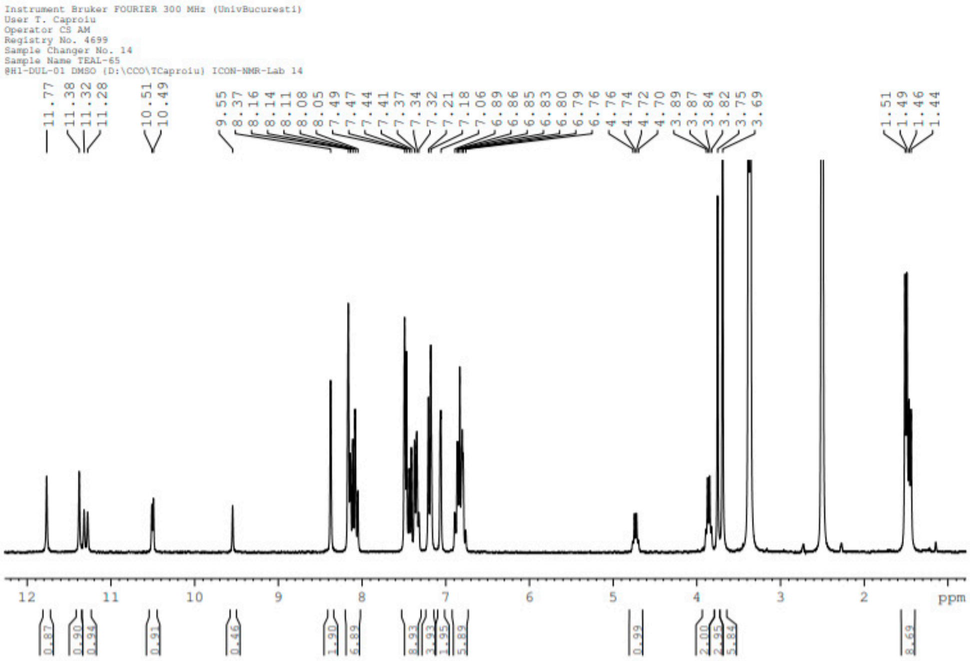

Figure S7. The ^{1}H-NMR spectrum of (*EZ*)-N'-(2-hydroxy-5-methoxy-benzylidene-(2*RS*)-2-(6-chloro-9*H*-carbazol-2-yl)propanehydrazide (**1d**)

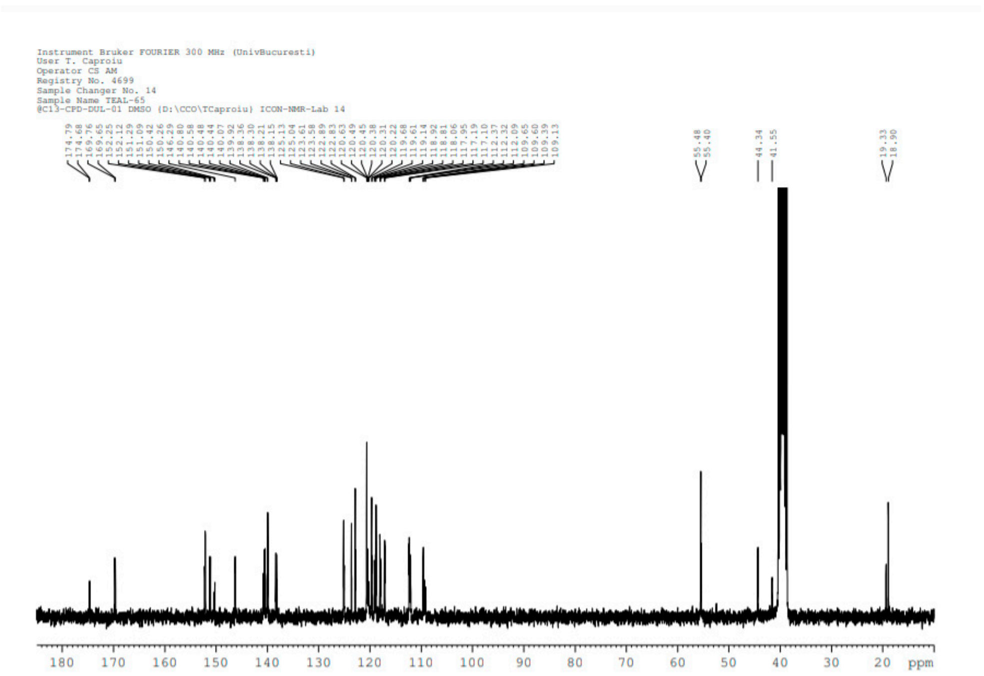

Figure S8. The ^{13}C-NMR spectrum of (*EZ*)-N'-(2-hydroxy-5-methoxy-benzylidene-(2*RS*)-2-(6-chloro-9*H*-carbazol-2-yl)propanehydrazide (**1d**)

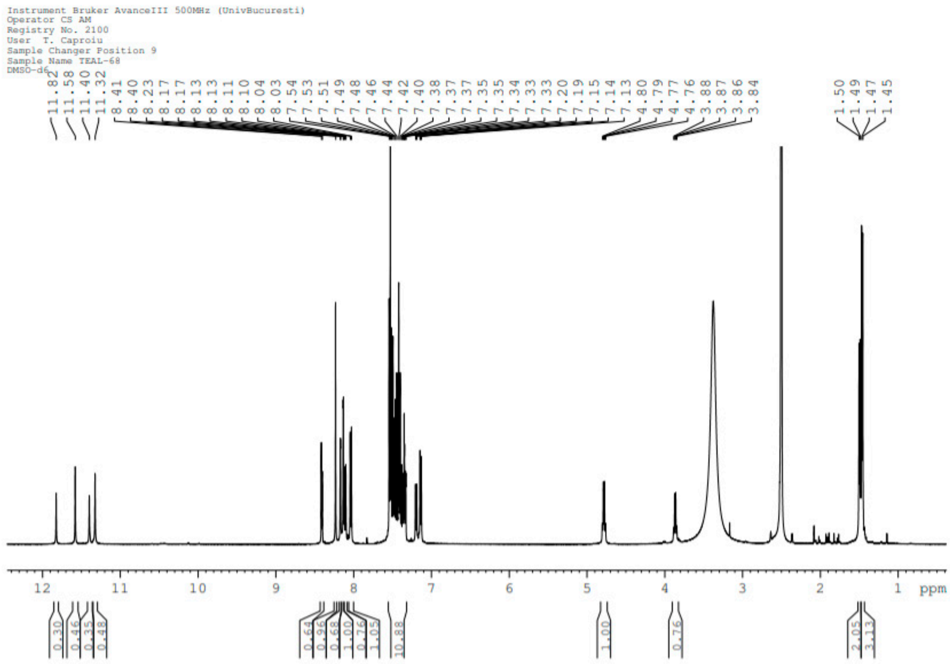

Figure S9. The ^{1}H-NMR spectrum of (*EZ*)-N'-(2,6-dichloro-benzylidene-(2*RS*)-2-(6-chloro-9*H*-carbazol-2-yl)propanehydrazide (**1e**)

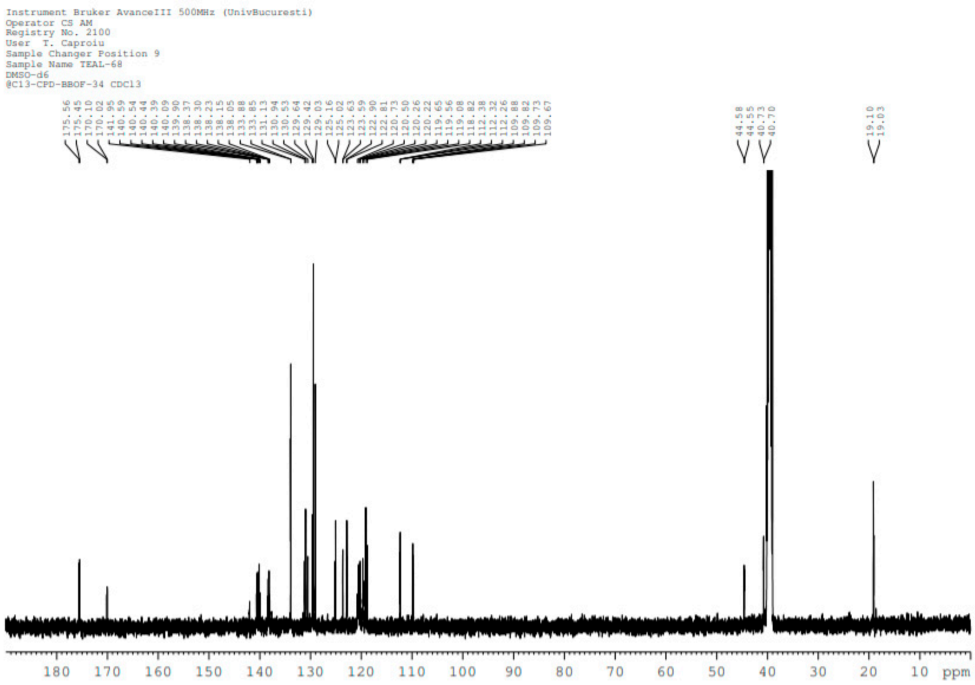

Figure S10. The ^{13}C-NMR spectrum of (*EZ*)-N'-(2,6-dichloro-benzylidene-(2*RS*)-2-(6-chloro-*9H*-carbazol-2-yl)propanehydrazide (**1e**)

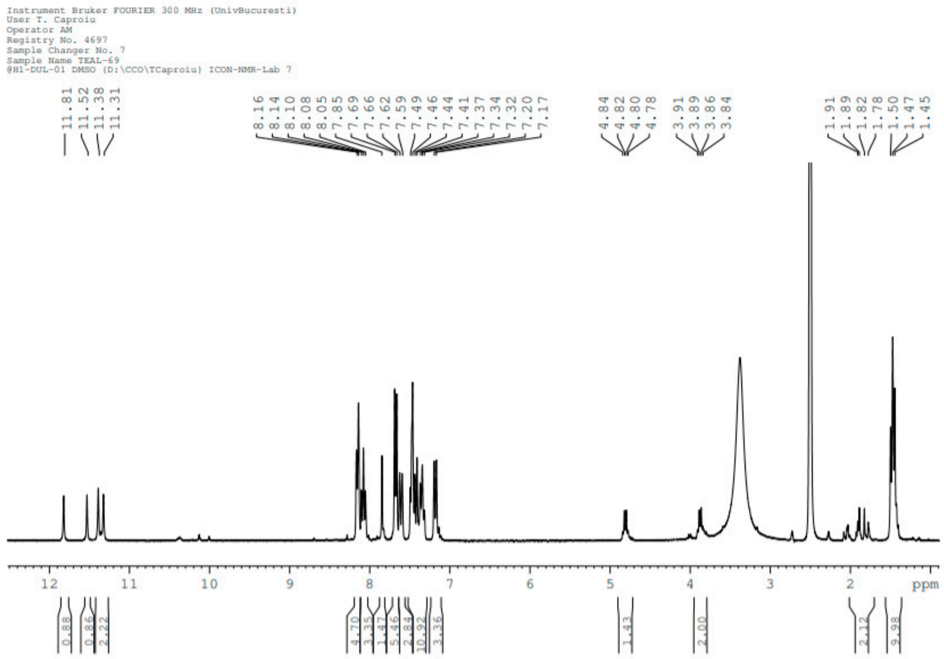

Figure S11. The ^{1}H-NMR spectrum of (*EZ*)-N'-(3,5-dichloro-benzylidene-(2*RS*)-2-(6-chloro-*9H*-carbazol-2-yl)propanehydrazide (**1f**)

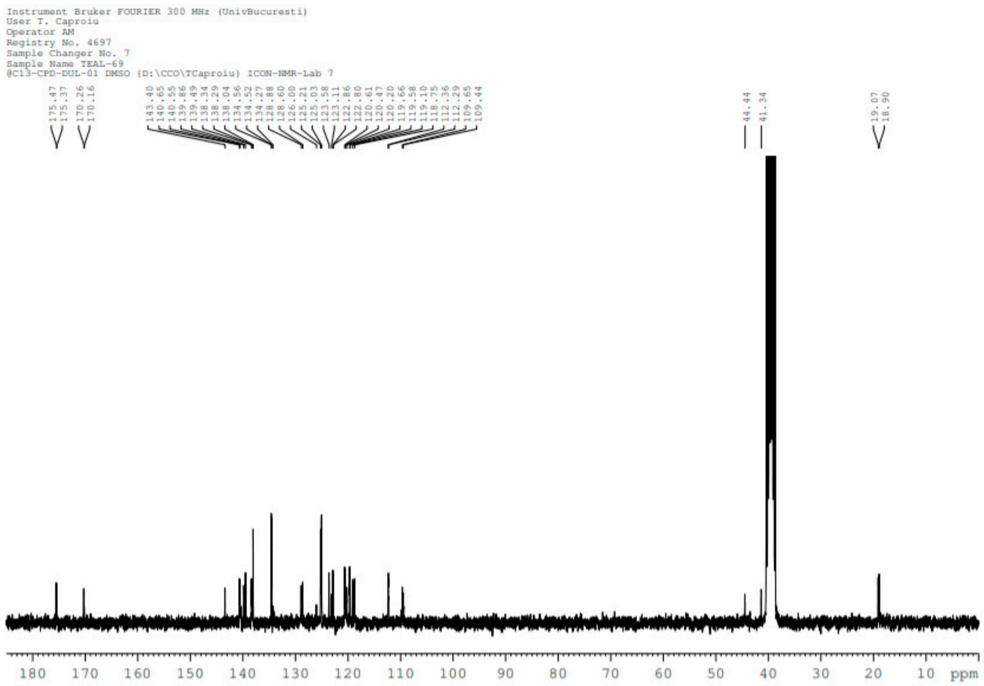

Figure S12. The ^{13}C-NMR spectrum of (*EZ*)-N'-(3,5-dichloro-benzylidene-(2*RS*)-2-(6-chloro-*9H*-carbazol-2-yl)propanehydrazide (**1f**)
